# Insights from the front line: uplifting stories of the COVID-19 pandemic through the eyes of the public health workforce in Iowa

**DOI:** 10.3389/fpubh.2025.1597941

**Published:** 2025-07-23

**Authors:** Heidi Haines, Amanda Sursely, Hanh Pham, Rylee Beltran, Daniel K. Sewell, Lina Tucker Reinders, Natoshia Askelson, Sarah Dixon, Kaitlin Emrich, Christine Estle, Michelle Lewis, Edith Parker, Jimmy Reyes, Laurie Walkner, Kelly Wells Sittig, Rima A. Afifi

**Affiliations:** ^1^College of Public Health, University of Iowa, Iowa City, IA, United States; ^2^Iowa Public Health Association, Des Moines, IA, United States; ^3^Iowa Primary Care Association, Des Moines, IA, United States; ^4^Black Hawk County Public Health, Waterloo, IA, United States; ^5^Jefferson County Public Health, Fairfield, IA, United States; ^6^Siouxland District Health Department, Sioux City, IA, United States; ^7^Department of Nursing & Public Health, College of Social & Behavioral Sciences, University of Northern Iowa, Cedar Falls, IA, United States; ^8^Iowa Cancer Consortium, Coralville, IA, United States

**Keywords:** COVID-19, public health workforce, SenseMaker, mixed-methods, rural health, mental health

## Abstract

**Introduction:**

SARS-CoV-2 was declared a pandemic in March 2020. Studies have characterized some of the negative impact of the pandemic on public health workers (PHW), but few have explored the strength and coping strategies used. Our study documents the experiences of PHW in Iowa during the pandemic.

**Methods:**

We used an innovative mixed methods data collection tool, SenseMaker^®^ to gather stories from PHW in Iowa between March and July 2022. Participants provided additional data about their story via structured follow-up questions. We conducted descriptive analyses of the quantitative questions. A qualitative thematic and sentiment analysis was applied to all stories.

**Results:**

The final sample size was 163 stories. PHW who submitted stories identified as mostly white (93.9%), and women (83.4%). Respondents were most likely to feel that in the story they told: the ability to act was out of their control; the strongest influence was from political considerations; decisions were made out of necessity; they wished for more focus on evidence-based public health. PHW were most likely to describe their emotional state at the height of the pandemic as frustration (61%), uncertainty (55%), and helplessness (50%). Those who felt in control were more likely to express positive emotions. Qualitative thematic analysis of these stories revealed 8 themes, highlighting facilitators (e.g., strong partnerships and collaborations) and barriers (e.g., politicization and controversial government response) to effective pandemic response.

**Discussion:**

The stories PHW shared powerfully illustrate the context of the pandemic in Iowa, a state that spent the least time under high stringency policies.

## 1 Introduction

SARS-CoV-2 was identified in December 2019 and rapidly declared a global pandemic by the World Health Organization on March 11, 2020 (1, 2). The first case of COVID-19 in the United States (US) was identified in January 2020 and until May 2, 2023, 106,678,503 people in the US were infected, and over 1.1 million people died as a result of COVID-19 (2). Excess mortality from COVID-19—i.e., mortality rates exceeding the expected rate for a year—ranged between 110 per 100,000 and 596 per 100,000 in the US, variability that was correlated with the pre-existing strength of the state's health system as well as the speed and comprehensiveness of the pandemic response (3). While the healthcare workforce was critical for COVID-19 hospital and ambulatory care, the public health workforce has been central to the US COVID-19 community response. Although there is no consistent definition of the public health workforce (4, 5), a common definition includes all those working in local, regional, state, and national health departments or other government public health agencies (63). In 2022, the public health workforce across the US ranged in size from 59 to 97 workers/10,000 population (or 35–53 FTEs/100,000) depending on region (6, 7). In contrast, in 2022, patient-care practicing physicians ranged in size across the US from 171 to 507/100,000 population depending on the State or District of Columbia (8).

### 1.1 Role of public health workforce and public health workers (PHW)

While the healthcare workforce was treating active COVID-19 cases, the public health workforce was running in parallel to provide prevention and mitigation efforts. The public health workforce assumed responsibility for testing, contact tracing, isolation and quarantine, community communication, and overall coordination while engaging in an unprecedented vaccination campaign (9). The role of PHW in pandemic response was crucial to reducing poor health outcomes, deaths, and disease burden in the US (10). Yet, despite these efforts, the public health workforce did not received the widespread recognition as essential or frontline workers as did healthcare workers (11, 12), even as their role shifted throughout the pandemic and came to mirror, supplement or overlap the tasks of healthcare workers (13).

While all PHW were impacted by the pandemic, roles and activities varied across the US. Generally, states in the South (e.g., Georgia, Mississippi, West Virginia) and the Midwest (e.g., Iowa, Michigan, South Dakota), had less stringent public policy responses to COVID-19 (14). States in the Midwest had higher infection rates than the US as a whole, whereas Southern states had highest COVID-19 death rates, both outcomes related to the less stringent mitigation policies in these states (15, 16). Rural communities faced additional challenges due to pre-existing conditions such as poverty, reduced access to healthcare, provider shortages, and high levels of chronic illness which placed significant strain on the healthcare system and public health workforce even prior to the pandemic (17).

### 1.2 Impact of the COVID-19 pandemic on the public health workforce

Several systemic factors pre-dating COVID-19 have been identified as contributing to the US public health system's response to COVID-19 and perhaps subsequently, the impact that the COVID-19 response had on the public health workforce. Historical and continuing disinvestment in public health has enabled infrastructural fragmentation, staffing shortages, and insufficient funding that have weakened the public health system (9, 10, 18). Alongside issues of staffing shortages, high turnover rates have resulted in a public health workforce with gaps in capability or experience (19).

The pandemic's impact on PHW has not been well studied (20). Stress, harassment, and mental distress have been found to be critical outcomes of the COVID-19 pandemic on PHW (12, 13, 21, 22). Misinformation and conspiracy perpetuated in the US media increased polarization and threats from community members (11, 23). Fifty seven percent of PHW in the US reported a minimum of 1 harassment incident (24) and 54% of PHW in Canada felt bullied, or harassed (25). These instances of harassment were associated with a higher risk of PHW leaving or intending to leave the workforce (25, 26). The pandemic also resulted in deteriorating mental health. In 2020, 66.2% of US PHW indicated they were burned-out (27), up from 23% prior to the pandemic (28, 29). PHW who felt bullied, threatened, or harassed were significantly more likely to report mental health symptoms than those who did not report these experiences (22).

Few studies have attempted to understand the strength and coping strategies among PHW through the COVID-19 pandemic. Physical activity, prayer or other spiritual practices, and social connection were associated with reduced reported mental health symptoms among US PHW (22). PHW also expressed feelings of hope that the increased visibility of public health in the US would improve funding, public support, and public awareness for the field in the future (30). Flexibility in duties, and location of work; as well as hazard pay and vacation time also improved the wellbeing of PHW (13, 22, 31).

### 1.3 The COVID-19 response in Iowa

The COVID-19 pandemic arrived in Iowa later than in other states (32), with the first reported case on March 8, 2020 (64). Iowa also had more COVID-19 deaths in rural areas than other rural areas in the country, at least partially attributed to the meat packing industry and its mismanagement of the pandemic, at great cost to workers (65). In Iowa, there are more meat packing jobs than the US average and they are located in rural areas (32). The response to the COVID-19 pandemic in Iowa was ranked 22nd in the nation (1 being the best response) based on indicators such as “progress in vaccinating residents, COVID-related hospitalization rates and health system stress, and COVID-related mortality through the end of March 2022” (3). Iowa was one of the states that spent the least time under high stringency policies (14). For example, Iowa did not issue a stay-at-home order, which has been attributed to an initial 30.4% excess in cases of COVID-19 compared to neighboring states (33) but did close schools for the spring semester of 2020 and closed restaurants, libraries, and fitness centers in March until mid-May 2020. Quarantine policies also evolved to differ from CDC recommendations, with guidance changing in September 2020 to not require quarantine if one was exposed to a COVID-19 case while both persons wore masks. Iowa's COVID-19 vaccination rate in 2021 was at 56%, with lower rates in rural areas (34, 35). While debate has arisen in the public health community about the cost/benefit ratio of some of the COVID precautionary policies (36), at the time of the pandemic, the public health workforce were following CDC guidance.

### 1.4 Aim of our project

Much of what we know about the impact of COVID-19 on the public health workforce comes from the Public Health Workforce Interests and Needs Survey (PH WINS) (37), the most complete national data set on the PHW experience during COVID-19. While this is a critical tool in understanding the PHW experience, it fails to completely capture the broad nuance of the public health workforce, particularly for smaller and more rural public health departments such as in Iowa, where only 4 health departments were included in PH WINS (38, 39). There is also a need for qualitative research on the impact of COVID-19 on PHW experiences.

This study aimed to document and characterize the COVID-19 response in Iowa by uplifting the experiences of PHW at the frontline, and to learn from these experiences to guide future response efforts.

## 2 Materials and methods

We used a mixed methods software platform to gather stories from PHW in Iowa. The Iowa Public Health Association (IPHA) and the Prevention Research Center for Rural Health (PRC-RH) at the University of Iowa partnered to conduct the project. A project planning committee consisting of individuals from academia, local public health departments and statewide health organizations, guided the design and implementation of the study. The planning committee's role was to help develop the story prompt and follow up questions, assist with pilot testing of the platform, and recruitment of participants, as well as interpretation of results, and writing of this manuscript.

### 2.1 Data collection tool

We used an innovative mixed methods data collection tool, SenseMaker^®^, that allows for visualization of patterns across participant-reported narrative stories (40). SenseMaker^®^ uses open-ended story prompts that allow participants to qualitatively share their experiences, and then gathers additional data about the story via structured follow-up questions (41, 42).

**Figure 1:**
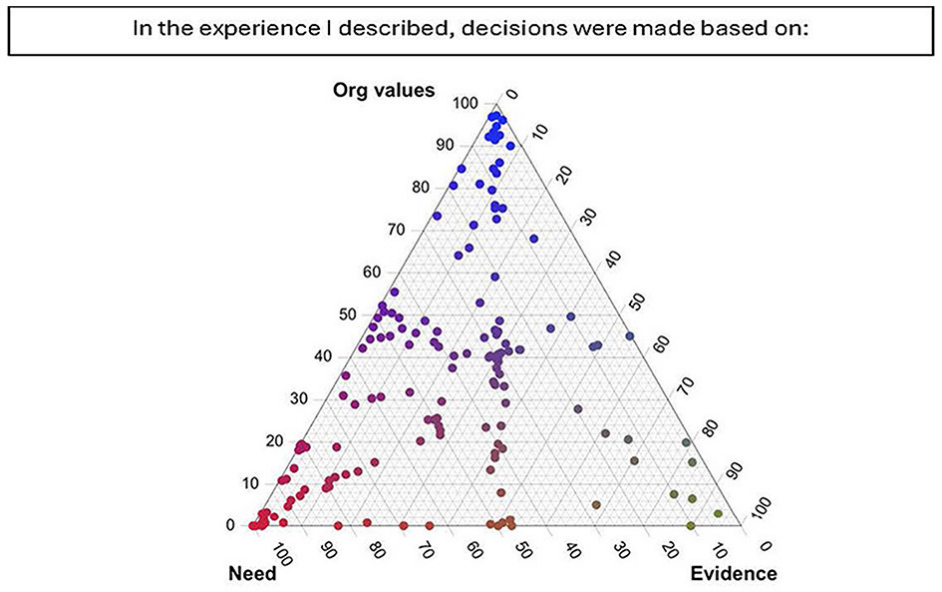
Ternary plot showing PHW perspectives on factor driving decisions in their stories. Respondents were able to place their cursor anywhere in the triangle. This plot shows that many placed their cursor at one of the corners indicating that variable to account for the majority of the decision. Others placed their cursor in between the line linking need and organizational values, indicating that both those variables equally accounted for the decision.

**Figure 2:**
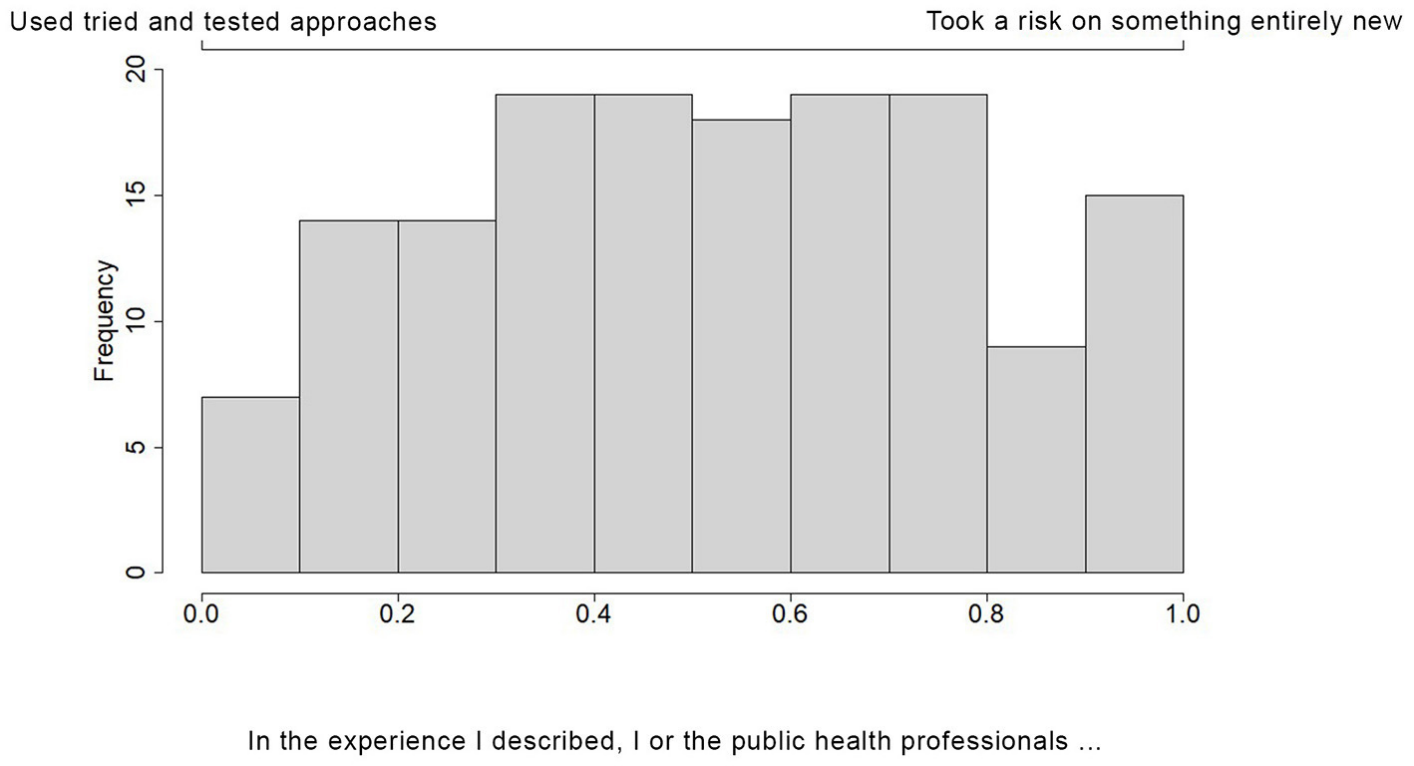
Dyadic bar chart showing PHW perspectives on whether the story they described included the use of tried and tested approaches, or took a risk on something entirely new. PHW were able to place their cursor anywhere along the 2-dimensional line. This graph shows the frequency with which PHW chose various positions on that line.

For our tool (Supplementary appendix A), participants were asked to share either a typed or voice-recorded story in response to the following prompt: “Imagine you are trying to explain to someone who does not work in public health how our public health system responded to the COVID-19 pandemic in Iowa. What would you tell them?” A series of follow-up questions were asked that related to the individual's perception of their story. All participants received the same set of follow-up questions. The first seven follow-up questions were ternary in nature (triads), where each individual's response was made by placing a point on a ternary plot; that is, each answer was a point in a 3-dimensional simplex. Each point in the 3-dimensional simplex corresponded to a composition, which is a mixture of three related variables that together sum to 100%. Each corner of the plot represented a condition where one variable constitutes the entire composition. The closer a point to a corner, the more dominant that variable is within the individual's response. Participants were able to move their cursor anywhere within the 3-dimensional simplex (e.g., triangle). Choosing one corner indicates that the answer to the question was only related to the corner variable. For more information on ternary plots and their interpretation, see the Supplementary material B, or for more details on this, as well as compositional data analysis, see Smithson and Broomell (43) or any standard text on compositional data analysis (43). Figure 1 gives an example along with all responses given as points. Responses to the subsequent 5 questions fell along a 2-dimensional simplex (Dyads); in these questions, participants were able to move their cursor anywhere along the 2-dimensional line. Figure 2 gives an example of the first such question along with all responses. The remainder of the follow-up questions were standard multiple-choice questions. At the end of the survey, participants could also choose to answer a series of three optional, open-ended questions that were unrelated to their original story, as follows: “How has this process of thinking about your experience felt?”, “Use the space below to share one insight you've had about the impact of the pandemic in your local community”, and “Imagine a new but equally virulent pandemic is beginning its spread. Given the experience of the past year and a half, what should we do differently this time around?”. Participants could elect to resubmit the survey with additional stories, and all stories were anonymous.

### 2.2 Data collection

A pilot test was conducted in Fall 2021 with 30 local PHW, invited by the project planning committee members. Individuals completing the pilot survey were asked to provide additional evaluation feedback on survey length, question clarity, and user interface. Incentives were provided which included a public health heroes t-shirt created specifically for this project, as well as a chance to enter a drawing for a $30 gift card. The pilot tool was adjusted based on the feedback, and the final version was approved by the project planning committee.

The full survey was distributed between March 2022 and July 2022. The invitation invited the ‘public health workforce' to complete the survey, using email listservs from the Iowa Cancer Consortium (1,700 individuals) and the Iowa Public Health Association (4,500 individuals). We refer to these respondents henceforth as PHW although we did not use any specific definition (except that they were not treating patients in clinical hospital settings), allowing respondents to self-identify as members of the public health workforce. The same incentives were provided for the full study as for the pilot (Gift card ratio: 1 for every 20 respondents).

### 2.3 Data analysis

**Figure 3:**
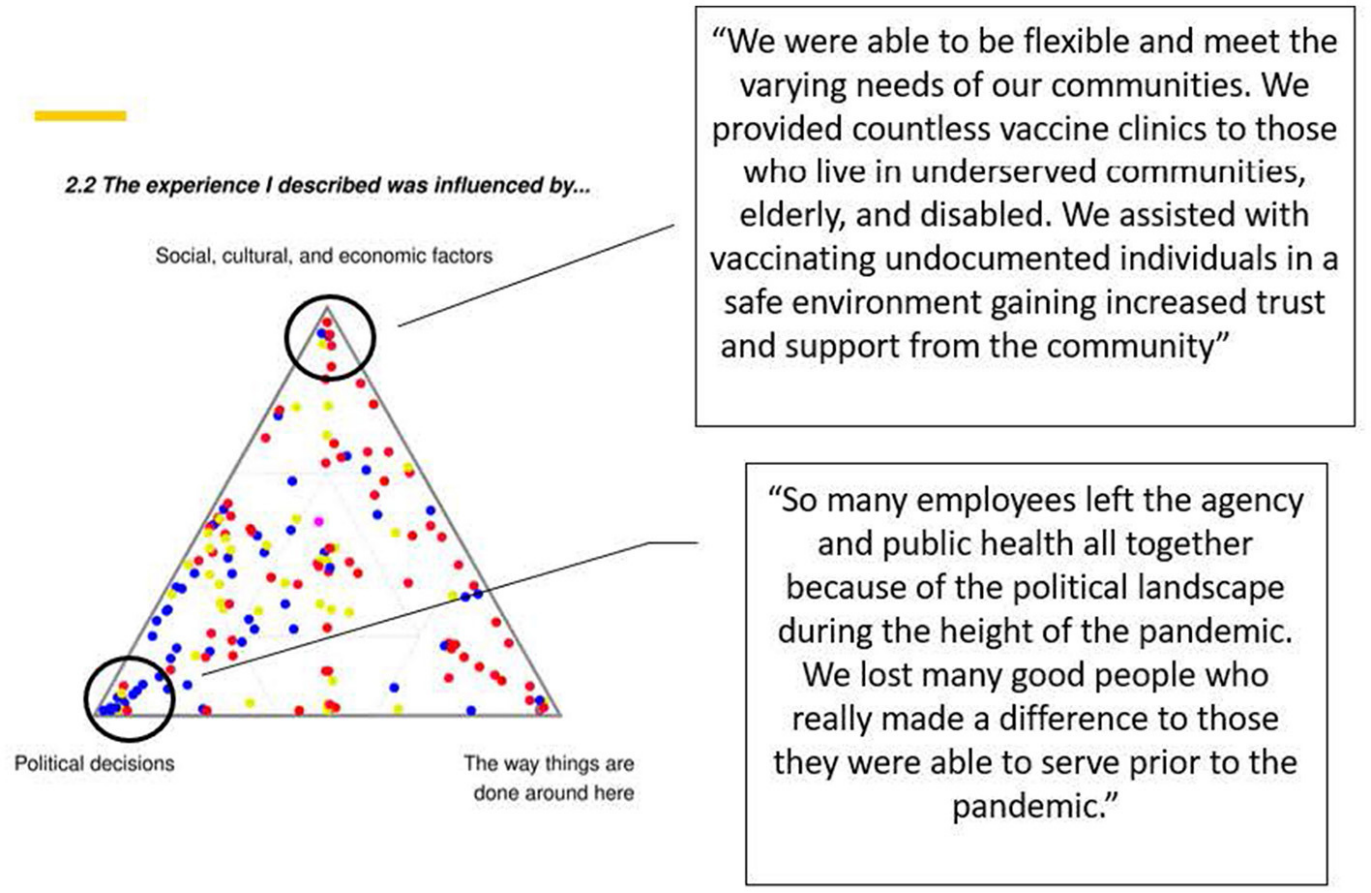
Ternary plot showing PHW perspectives on factor driving the experience of their stories. Stories were linked to quotations drawn from datapoints at the axes, with two examples shown here.

**Quantitative:** We conducted descriptive analyses of the questions reported via the SenseMaker^®^ tool and the follow-up multiple-choice questions regarding PHW perception of their story. For questions allowing responses on a simplex, we calculated the mean of each component within the composition. We then wished to assess the relationship between these dyadic and triadic variables with several multiple-choice questions which allowed PHWs to choose up to three emotions that accurately reflected their current emotional state and their feelings at the height of the pandemic. Standard correlation analyses are not appropriate for compositional data, instead, “Compositional data analysis is frequently associated with applying an appropriate transformation first, and then employing the standard statistical methodology as usual” (44). Hence, we applied a centered log ratio transformation to our compositional variables—thereby mapping the data from a constrained simplex space to an unconstrained Euclidean space—and then applied correlational analyses to them, as has been common practice since Aitchison's 1986 monograph. Because both our compositional variables and our multiple-choice questions were multivariate, we applied the long-established canonical correlation analysis (CCA) (45). CCA aims to determine the correlation between two sets of variables by finding the linear combinations of each set that are most highly correlated with each other. See the Supplementary material B for a brief overview. **Qualitative:** A randomly selected subset of 20 (out of a total of 163) stories were used to develop relevant codes for qualitative thematic analysis. The resulting codebook was used for the remainder of the stories and open-ended optional questions. All qualitative coding was conducted in Dedoose (46). Story responses and quotes were then linked to the triads, dyads, and multiple-choice questions to compare thematic differences across quantitative response groups. For example, we compared the themes in the stories of those who indicated that they viewed their story positively vs. negatively in the multiple choice question asking about how they felt about the experience/story they shared. Figure 3 provides a visual example of how the qualitative stories were integrated in with these data, highlighting two quotations drawn from stories on polarized axes of a ternary plot.

In addition to this more traditional method of qualitative analysis, we also performed a sentiment analysis (47) on the story text to gain a deeper understanding of the emotional perception of the pandemic among PHW. For each story, we calculated the percentage of sentences with positive, negative, and neutral sentiment. The sentiment of a sentence is an unbounded value computed using the sentimentr package in R42 (48). A zero value signifies neutral sentiment, while a positive or negative value indicates corresponding sentiment. The sentiment value calculation involves assigning scores to individual words based on their general sentiment while considering valence shifters, such as negators and amplifiers. Valence shifters are words that can alter the sentiment; for example, the word “not” can reverse the emotional tone of subsequent words.

The Institutional Review Board of the University of Iowa approved this research as exempt on 6.7.22. All participants consented to complete the survey.

## 3 Results

We collected a total of 179 stories and excluded 14 stories that were clearly from a clinical hospital treatment setting, and 2 stories that did not have any valid text. The final sample size was 163 stories. Table 1 shows the descriptive statistics of PHW with stories included in this analysis. PHW who submitted stories identified as mostly white (93.9%), women (83.4%), and aged 36 and older (75.5%). Ten stories are displayed in Supplementary appendix C, selected intentionally as examples to showcase the broad range of experiences and feelings expressed in the greater sample. Tables 2 and 3 show the mean composition of follow-up triadic and dyadic questions, respectively, reported via the SenseMaker^®^ tool. Respondents tended to feel that—for the story they told: the ability to act was out of their control; the strongest influence was from political considerations; their community was most impacted; decisions were made out of necessity; responsibility lay with government and large organizations; they wished for more focus on evidence-based public health; PHW took the initiative; PHW reached out to formal channels; public health systems prioritized fast response and action over caution; and public health systems reached underserved populations. The following excerpts from submitted stories support several of the above results:

“We as a department had very little time to prepare for this pandemic and we had to adapt quickly to ever changing data and guidance from the CDC, FDA, and the WHO at a national level, but then we also had to be up to date with all of the state and local mandates, shutdowns, etc. It was very challenging to keep up with all of the information coming from so many different sources and we are just here trying to help our community stay healthy, while taking a lot of abuse from those that do not agree with what is going on. Every day we received so many phone calls from different people in the community, confused, angry, wanting help, and there was only so much we could do.”

“One minute we were watching the world get hit by COVID-19; the next it officially arrived in our community. As a local health department, we immediately banded together to identify guidance for the community, establish data tracking mechanisms and workflows, and band together all health partners. While not perfect, we worked rapidly to follow changing state and national guidance and many times learned of changes as the public did at state press conferences.”

**Table 1:** Demographic of PHW who submitted stories.^*^

	**Overall (*N =* 163)**
**Age**	**N (%)**
18–25	4 (2.5%)
26–35	32 (19.6%)
36–45	41 (25.2%)
46–55	43 (26.4%)
56–65	29 (17.8%)
Over 65	10 (6.1%)
Missing	4 (2.5%)
**Gender**	
Man	22 (13.5%)
Woman	136 (83.4%)
Missing	5 (3.1%)
**Transgender**	
I prefer not to answer	2 (1.2%)
No	161 (98.8%)
**Race**	
Asian	2 (1.2%)
White	153 (93.9%)
Prefer not to answer	8 (4.9%)
**Region** ^a^	
1	23 (14.1%)
2	10 (6.1%)
3	18 (11.0%)
4	11 (6.7%)
5	20 (12.3%)
6	81 (49.7%)

^*^percentages may be >100% due to rounding.

^a^Map of regions can be found here: https://hhs.iowa.gov/media/13038/download?inline.

**Table 2:** Average compositions in questions about PHW general reflection of the experience.

**PHW reflections of their story**	**Component 1**	**Component 2**	**Component 3**
**In the experience I described…**	29% (I had control over the action)	33% (I was able to act)	38% (the ability to act was out of my control)
**The experience was influenced by …**	33% (social/cultural/economic factors)	40% (political decisions)	27% (the way things are done around here)
**There was an impact on …**	29% (myself/loved ones)	39% (my community)	32% (my organization)
**Decisions were made based on …**	35% (organizational values and mission)	22% (evidence)	43% (necessity)
**Responsibility lay with …**	24% (individual actions)	33% (collective behavior and organization)	43% (government and large organization)
**I noticed people worked with … partners**	43% (existing)	40% (new local)	17% (new national, state, or federal)
**I wished for more …**.	25% (access and inclusion)	33% (understanding and empathy)	42% (focus on evidence-based public health)

Darker colored cells indicate higher values, implying greater dominance of the corresponding component in a three-part composition.

**Table 3:** Average compositions in questions describing the responses of PHW and PH system to the crisis.

**PHW responses to dyadic questions**	**Component 1**	**Component 2**
**I or PHW …**	48% (used tried and tested approaches)	52% (took a risk on something entirely new)
**I or PHW …**.	60% (took the initiative)	40% (waited for instructions)
**I or PHW reached out to …**	45% (informal networks)	55% (formal channels)
**PH systems prioritized …**	63% (fast responses and actions)	37% (caution and took time to reach a decision)
**PH systems reached …**	38% (same people as before)	62% (underserved populations)

Darker colored cells indicate higher values, implying greater dominance of the corresponding component in a two-part composition.

**Figure 4:**
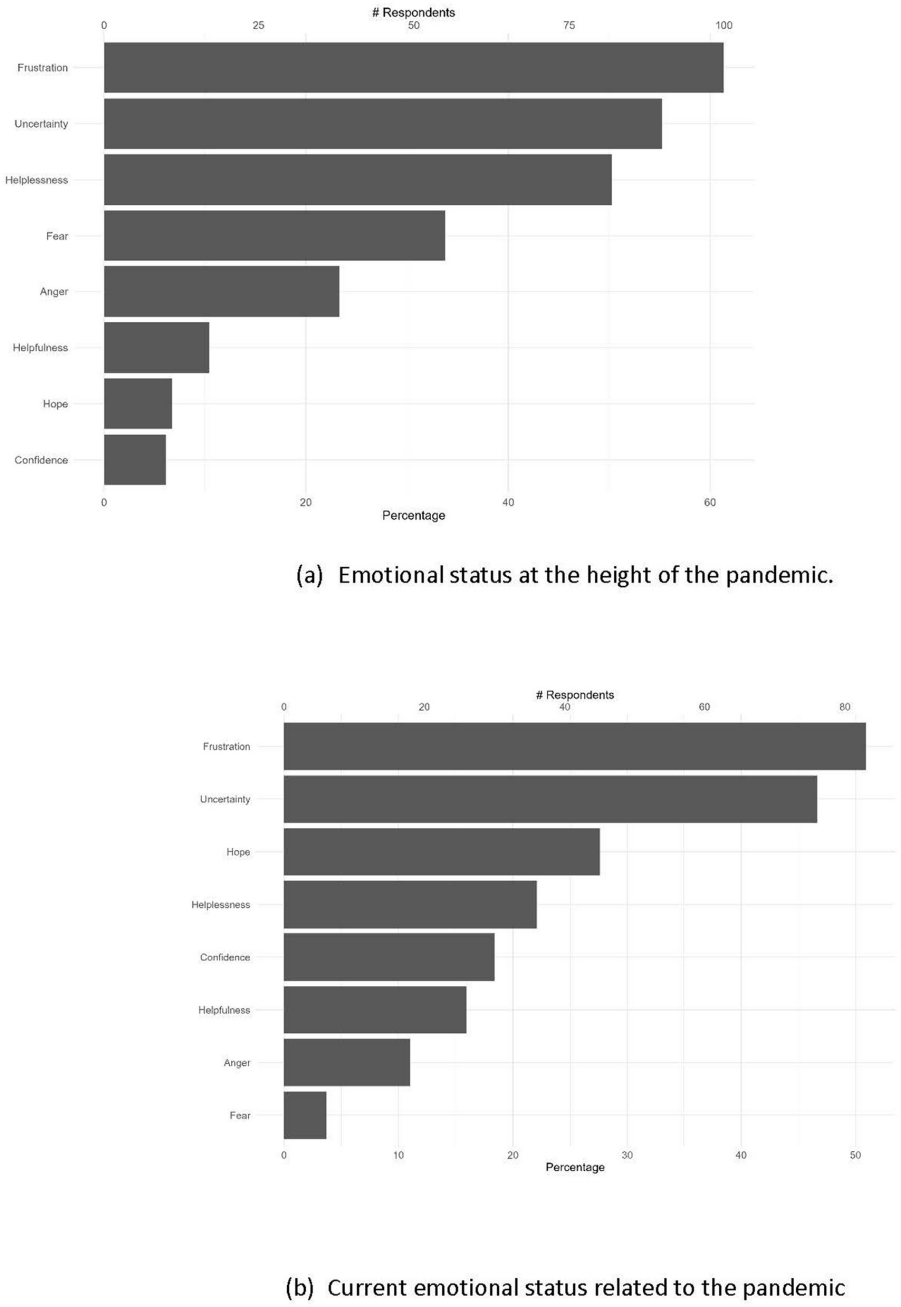
Percentage of PHW that selected these respective feelings as best describing their emotional status related to the pandemic's impact of their work in public health **(A)** at the height of the pandemic—in Winter 2020-Spring 2021, and **(B)** at the time of the survey—in Spring/Summer 2022. PHW could pick up to 3 feelings.

When PHW were asked to select up to three feelings that best described their emotional state at the height of the pandemic (which we defined in the survey as “Winter 2020–Spring 2021”), the most frequently reported feelings were frustration (61%), uncertainty (55%), and helplessness (50%) (see Figure 4A). It is worth noting that not all reported feelings were negative; a small percentage of PHW mentioned feelings of helpfulness (10%), hope (7%), and confidence (6%). These varied emotions were exemplified in the stories:

“As each week progressed, health professionals across disciplines would call and harass our staff… It is shameful how local public health staff were treated.”

“We absolutely, without a doubt, 100 percent would not have been successful at the mass vaccination clinics without all of these amazing people! They are all heroes during the vaccination campaign and deserve recognition. So, I want to say “THANK YOU!”.

PHW also experienced a high level of uncertainty regarding the safety of themselves, their loved ones, and their communities. The percentages of PHW selecting “uncertain or very uncertain” were 57%, 66%, and 77%, respectively (data not shown).

“We had to hear how sick people were and not able to help them. Our staff quietly shed many tears, all while worrying about the health and safety of their family and themselves.”

In PHW perspectives, COVID-19 mitigation strategies such as masks and testing were mostly available at a cost (8%), available for some (49%) or available for all (37%) (data not shown).

When asked to describe their feeling at the time of completing the survey in Spring-Summer 2022 (as opposed to at the height of the pandemic), we found similar patterns in PHW current feelings related to the impact of the pandemic, with frustration (51%) and uncertainty (47%) being the most cited emotions (see Figure 4B). However, there were more mentions of positive feelings, such as hope (28%), confidence (18%), and helpfulness (16%) (see Figure 4B). Despite this, the persistent stress and trauma experienced by PHW at the height of the pandemic can be seen through their response to the optional question “How has this process of thinking about your experience felt?”, with answers such as “Thinking about our response during the highest point of the pandemic makes me very emotional” and “Thinking back over my COVID experience to answer these questions has made me angry and frustrated all over again”.

**Figure 5:**
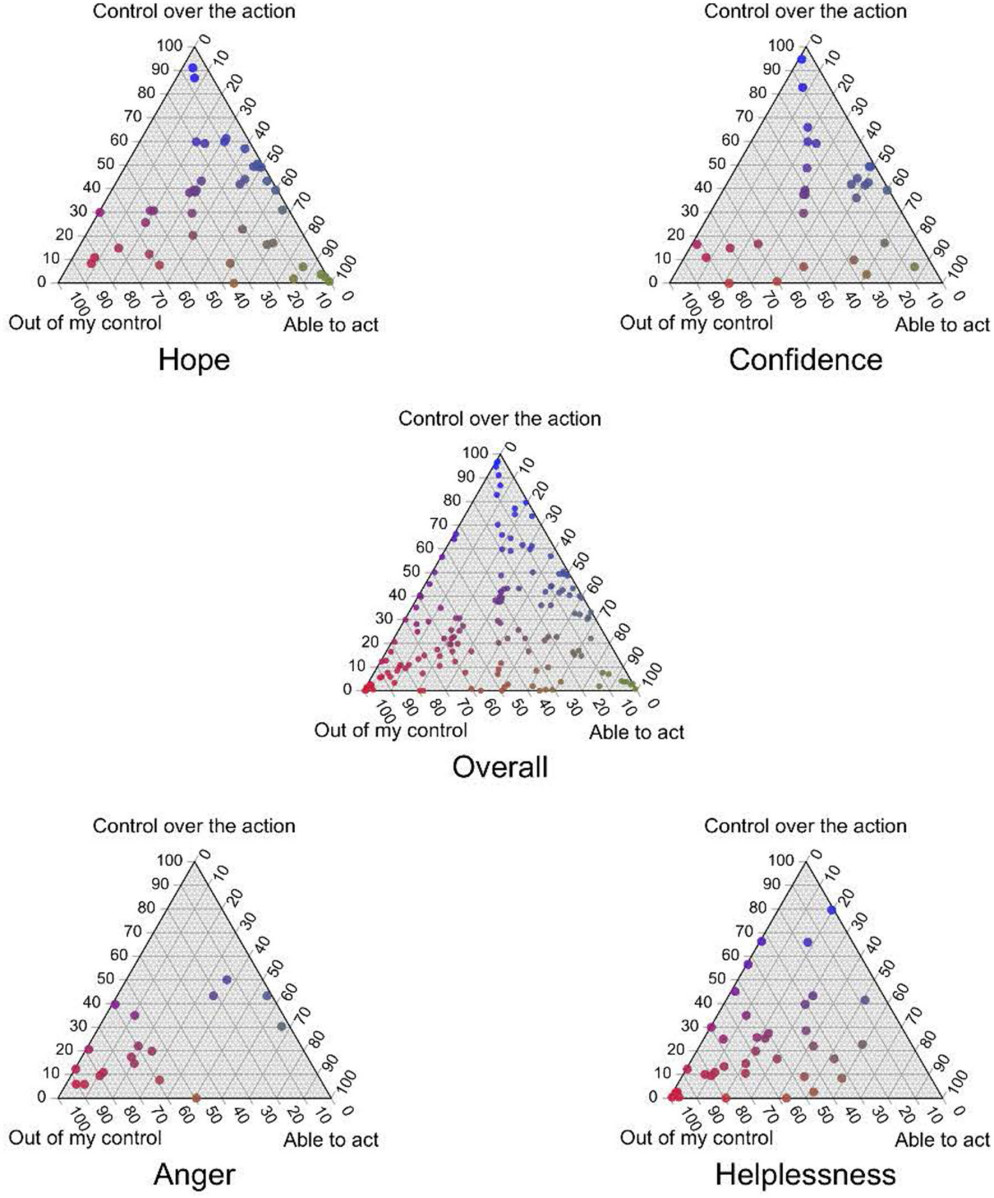
The relationship between public health workers' (PHW) perceived control in responding to the pandemic and their current pandemic-related emotions; each ternary point represents a unique story. The “Overall” central plot aggregates data from all participants, while the surrounding plots represent subsets of respondents who expressed certain emotions.

Table 4 presents the canonical correlations between the triadic/dyadic responses and PHW current (in Spring/Summer 2022) pandemic-related emotions and feelings at the height of the pandemic (Winter 2020–Spring 2021). Most of the coefficients are below 0.3, suggesting a weak correlation. We delved deeper into the most pronounced correlation, which is between PHW perceptions of control in pandemic response and their current emotion related to the pandemic. Figure 5 illustrates the relationship; each point on a ternary represents a story. The central plot, labeled “Overall,” includes all respondents, whereas the peripheral plots exclusively include respondents who reported specific emotions. Out of eight possible emotions, we report only those that exhibit a discernible pattern. Most participants who felt hopeful and confident also perceived themselves as having control and the capability to take action. Conversely, those who felt angry and helpless generally saw themselves as lacking control in their narratives. Similar plots examining other correlations with coefficients >0.3 are available in Supplementary appendix D.

**Table 4:** Canonical correlation coefficients between triadic/dyadic and PHW's current feelings related to the pandemic and feelings at the height of the pandemic.

**Triadic question response options**	**Current**	**At Pandemic**
***Triadic responses***		
I had control over action | was able to act | was out of control	0.41	0.28
**Experience influenced by** social, cultural, economic factors | political decisions | the way things are done	0.36	0.20
**There was an impact on** myself/loved ones | my community | my organization	0.26	0.25
**Decisions were based on** organizational values/missions | evidence | necessity	0.20	0.24
**Responsibility lay with** individuals | collective behavior and organization | government and large organization	0.27	0.29
**People worked with** existing partners | new local partners | new national, state, or federal partners	0.23	0.33
**I wished for more** access and inclusion | understanding and empathy | focus on evidence-based public health	0.31	0.25
***Dyadic responses***		
**I or PHW** used tried and testes approaches | took a risk on something entirely new	0.29	0.19
**I or PHW** took the initiative | waited for instructions	0.17	0.29
**I or PHW reached out to** informal networks | formal channels	0.18	0.28
**PH systems prioritized** fast responses and actions | caution and took time to reach a decision	0.26	0.23
**PH reached** same people as before | underserved populations	0.12	0.33

Each set of feelings consists of eight distinct emotions, such as frustration and hope. The coefficient ranges from 0 to 1, where 0 indicates no linear relationship and 1 indicates a perfect linear relationship.

**Figure 6:**
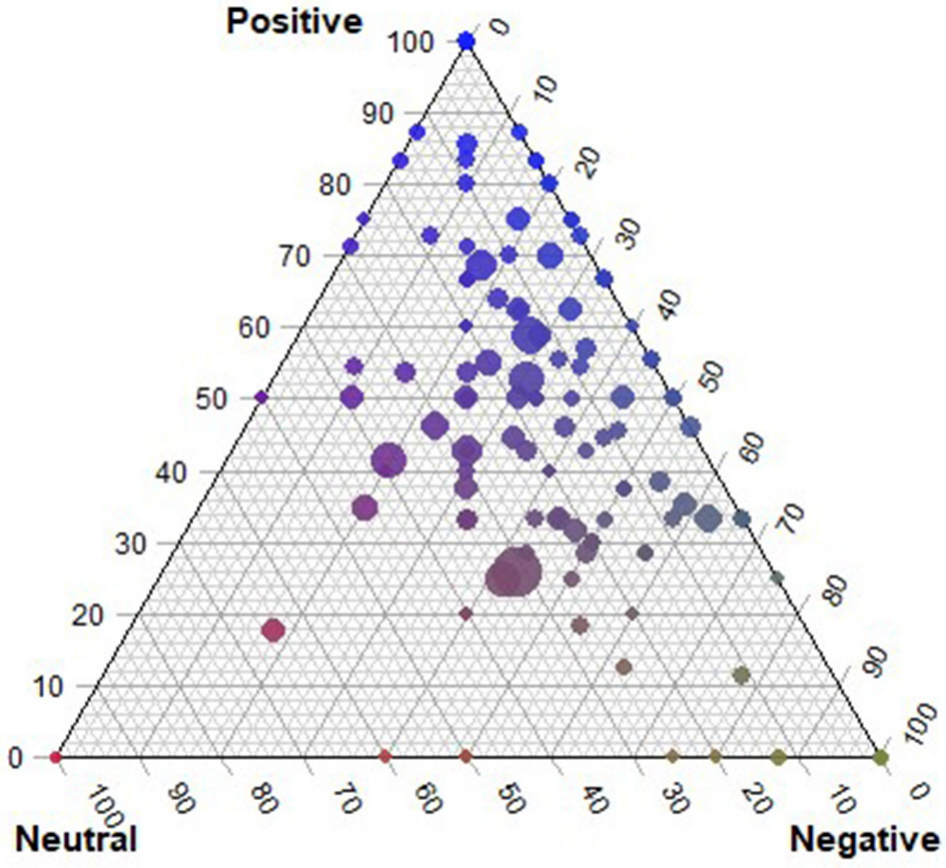
Compositions of neutral, positive, and negative sentences in each story. The size of each circle corresponds to the number of sentences in a story.

Figure 6 shows the results of the sentiment analysis of the story text, a computational linguistic method of assigning emotions (as opposed to asking directly as we did in the survey). Each point corresponds to a story, the location corresponds to the proportion of sentences that are positive, neutral, or negative, and the size of the point corresponds to the number of sentences in the story. The closer stories are to the top of the triangle, the more positive; the closer to the bottom left, neutral; and the closer to the bottom right the more negative. From this, we see that few stories are predominantly neutral in sentiment. Regardless of the length of the story, most comprised of more positive rather than negative and neutral sentences.

Qualitative thematic analysis of these stories revealed 8 themes. When summarized, they highlighted the facilitators and barriers to an effective pandemic response. Facilitators to an effective response included strong partnerships and collaboration, adaptable approaches involving innovative thinking, timely and effective communication, and an emphasis on equity in resource and service allocation (Table 5). Conversely, barriers to an effective response were evident in the politicization and controversial government response, lack of communication exacerbated by changing guidelines, conflicting information, mental health burden, workforce burn-out, lack of adequate resources such as staff and PPE; overwhelming workloads, and the public's varied perceptions of the pandemic (Table 5).

**Table 5:** Facilitators and barriers to an effective pandemic response, drawn from thematic analysis of *n* = 163 stories shared by public health officials, in response to the prompt “Imagine you are trying to explain to someone who does not work in public health how our public health system responded to the COVID-19 pandemic in Iowa. What would you tell them?”.

**Facilitator**	**Quotation (verbatim)**
Strong partnerships and collaboration	More than ever, public health had to rely on partnerships. We recognized early that we could not continue to support the work of contact tracing in our community as well as plan and implement vaccination clinics without help. As a result, in September 2020, I pulled together a group of health care partners from the hospitals and federally qualified health center to determine how we could handle the clinics in our community. We ended up expanding the group to include EMS and we met weekly (and continue to do so) to talk about how to allocate vaccine, how to staff clinics, etc. We established an MOU with the partners and there were many times when the clinics we hosted in the community were staff with more partners than Health Department staff. The energy and support these partners brought to the efforts were essential in bolstering our tired workforce!
Adaptable approaches involving innovative thinking	Working in public health during the beginning of the pandemic was difficult to say the least, but the silver lining was an opportunity to be creative and innovative in our various health programs. We were able to move our programs onto virtual platforms in the form of zoom classes, podcasts, and educational videos.
Timely and effective communication	Once vaccine became approved/available, we targeted qualified groups to receive their vaccine in a timely manner. Our team assured keeping up with the latest information so that we could be a trusted, reliable resource to our local medical community as well as citizens. When subsequent doses/boosters were due, our team worked to recall those who had previously received vaccine from our agency as well as utilize media/trusted citizens to outreach factual information in the flurry of rumors/fear.
Equity in resource/service allocation	Part of what we did was finding how and where to reach people in our community with current information as it was available. We took into account ways to reach our linguistically and culturally diverse population, and also considered literacy levels. We made contact tracing calls in both English and Spanish, many of them being evening or weekend calls. We created audio and video productions regarding COVID-19 in English, Spanish and Mam (a Guatemalan dialect). Materials were shared with workplaces, churches, businesses, social media, etc.
**Barrier**	**Quotation (verbatim)**
Politicization and controversial government response	We are not equipped to experience so much loss, grief and fear. Outside public health in business worlds, politics and even schools, COVID was at times mocked, reduced, minimized while each day I returned to my desk to see more death and suffering with the people I connected with. The disconnect and us vs them mindset with the politicization of public health continues to leave a distrust that is felt in community partnerships today.
Lack of communication	Lack of communication from the Governor's Office with state agencies and with local public health departments. This includes making public health decisions without input from IDPH or local PH partners. When the Governor issued a new proclamation, we would contact the state agency for clarification and we were usually told that they had already contacted the Governor's Office for clarification. By the time the Governor's Office replied, the agency revised its FAQ's, had them reviewed by Governor's Office and sent them out to the local PH Departments. the Governor would issue a new proclamation.
Changing guidelines and conflicting information	I feel as if our state did not have a central good system to relay messages about the pandemic. The messages were confusing and mismatching. The state did a poor job of providing information out to local county public health departments and the general public was very confused on where to get reliable information.
Mental health burden	We had staff and volunteers answering call center phone calls from people wanting vaccine who were not yet eligible who were screaming at our personnel on the other end of the line. We had to hear how sick people were and not able to help them. Our staff quietly shed many tears, all while worrying about the health and safety of their family and themselves.
Workforce burn-out	We were working crazy hours and it all felt very important, so it was a little energizing and fun sometimes, in the right room and in the beginning. Then, things turned really weird with a lot of people quitting, resigning or being forced to resign. Communication with the public was a mess, and things became very politically charged. People burned out and felt helpless and hopeless.
Overwhelming workloads	Immediate response. We went straight into the office to discuss how we were going to proceed once we received notification of our first positive case in the county. It was a Saturday. From that point on we worked 7 days a week trying to stay caught up with the ever changing updates and requirements.
Public's varied perception of the pandemic	The situation was becoming more political by the day and the public had extreme viewpoints and opinions for and against what we were trying to accomplish (saving lives).

The optional question “Imagine a new but equally virulent pandemic is beginning its spread. Given the experience of the past year and a half, what should we do differently this time around?” was answered by 82% of participants. PHW most commonly, and equally advocated for better, more timely communication, and a focus on evidence-based messaging rather than politics. Statements similar to “Communication, communication, communication from the top down” appeared in 36% of responses as did sentences like “Focus on the science instead of politics”. Those two messages were consistently reinforced, along with smaller percentages of individuals advocating for better resource allocation, increased funding and staffing, and better preparedness plans. As an example, the response “Focus on the science instead of politics. Do a better job of communicating with the public early on as to what they can expect. Work with a variety of partners to increase buy-in to public health mitigation strategies. Have access to more funding sooner (for staff and systems). Overall, everything needs to be more proactive than reactive. During this pandemic it often felt like public health was a step behind rather than a step ahead.” nicely summarizes several themes reflected in many answers.

## 4 Discussion

The results of this study served to document and characterize the COVID-19 response in Iowa by uplifting the experiences and stories of PHW at the frontline of the response. Here we focus on some of the main results that are supported by literature.

The rallying call of PHW focused on imploring the use of evidence-based practice and science, rather than politics; and of clear communication of that science. This call was supported by many analyses of the pandemic mitigation and outcomes in the US, which also indicate the difficulty of separating out each of these facts; i.e., politicization of the pandemic lead to failures in clear communication, and lack of reliance on evidence. The politicization of the pandemic has been described as “baked into the context of the emergent coronavirus” (49). New pandemics often result in less clear communication due to the limited knowledge, and the public are often likely to rely on the media or narratives of political elites (50). The politicization of the pandemic, which contradicted clear risk communication guidance, therefore fueled misinformation (49). Improved communication also emerged as one of several recommendations that 99% of experts agreed as necessary to end the COVID-19 threat (51). Analyses of risk communication strategies and messages related to the COVID-19 pandemic in the US further supports the inadequacy or failure of this communication (50, 52–54). Comparing the response to the pandemic to the guidance provided by the Center for Disease Control and Prevention (CDC's) Crisis and Emergency Risk Communication (CERC) guidance (55) results in the following conclusion: “the United States has both excelled and failed at all levels of government and public health authority” (50).

Strong partnerships and collaborations emerged from our respondents' stories as a critical facilitator of effective responses. Partnerships were also noted as critical to meet needs of the COVID-19 response in a report by the US Department of Health and Human Services (20). Teamwork and workplace camaraderie emerged also as an important protective factor for PHW mental health during the pandemic (30). Teamwork has been identified as one of the core principles that guide the competencies for disaster preparedness and response (56). Our respondents also highlighted the importance of adaptive and innovative action as a facilitator to effective responses, which confirms other literature (30, 57, 58).

Respondents noted that barriers to effective response included burn-out and mental health concerns. Studies conducted with PHW have found similar concerns; surveys inquiring about burn-out of PHW early in the pandemic (Spring–Fall 2020) found that 66.2% of US and 80% of Canadian PHW indicated they were burned-out (25, 27). In addition, in Spring 2021, 30% of US PHW reported symptoms of anxiety and of depression (21) and 56% of PHW reported one or more symptoms of PTSD (49); while in Fall 2022, 22% and 26% of Canadian PHW reported anxiety and depression symptoms, respectively (25). Our study added unique insight to the mental health of respondents, noting that those who felt in control and able to take action were more likely to feel hopeful and express positive emotions, whereas those who felt that action was not in their control were more likely to express negative emotions. Understanding the environments conducive to PHW sense of control, and ability to carry out their role is critical to supporting the health and wellbeing of this workforce.

Additionally, the mixed methods approach we use centered storytelling as a key component. “Stories are the fundamental way for… making sense of lives and coping with difficult experiences” (59), p. 83. Storytelling and narrative sense-making has been found to improve wellbeing (60, 61); our respondents noted the cathartic effect of sharing and analyzing their stories. Storytelling also allows for a much more expansive understanding of the issues, the context, and the realities of participants, thereby highlighting nuance and complexity of any issue, and “reveals insights not necessarily accessible by other means” (62), p. 11. Our respondents offered up deep insights, joys and pains of the COVID-19 pandemic in Iowa, and their analysis of their own stories provided guidance for key factors in pandemic preparedness and response going forward.

## 5 Strengths and limitations

This study has several limitations. The data collection relied on self-reported narratives, which may introduce bias and limit the generalizability of the findings. It is unclear what the response rate was; although we sent out to the listservs of the Iowa Public Health Association and the Iowa Cancer Consortium (combined *n* = 6,200), some of the same individuals could be in each of those listservs. Also, we invited those that received the invitation to the survey to share it with others. Irrespective, we received only 163 stories. Given this is a mixed methods study, 163 is significant as a qualitative sample size, though rather small from a quantitative methods perspective. Yet, this sample size, while providing rich qualitative insights, may not fully represent the broader PHW population in Iowa or other regions. The use of SenseMaker^®^ as a mixed methods tool, while innovative, also presents challenges in standardizing and interpreting complex narrative data. Finally, the study's focus on Iowa may limit the applicability of the findings to other states with different public health infrastructures and pandemic responses.

Despite these limitations, the study has several strengths. The focus on Iowa highlights the impact of the pandemic in less represented regions, as well as contributing to greater comprehension of regional disparities. Given that Iowa is a mostly rural State, our results provide insight into the impact of the pandemic for a rural health workforce. The mixed methods approach provided a combination of numbers and stories, nuancing the understanding of the pandemic's impact. Finally, our study adds to the literature on the experiences of PHW in the COVID-19 pandemic and contributes to the ongoing dialogue on improving public health response efforts.

## 6 Conclusion

The stories PHW shared powerfully illustrate the context of the pandemic in Iowa, a state that implemented few mitigation measures. Stories highlight the severe strain on the mental health of public health professionals during the pandemic and more currently, including feelings of frustration and lack of appreciation. However, the stories also uplift the solidarity and camaraderie developed with others in the public health field, along with innovative solutions developed to respond to this crisis. Participants found telling their stories in this format therapeutic and welcomed the opportunity to share, with the goal of enhancing future response. The results of this study provide deep insight into how to support the continued healing from the COVID-19 pandemic, as well as how to respond to a future pandemic. Key factors that were highlighted include the critical importance of partnerships, effective risk communication principles and techniques, and the reliance on evidence rather than politicization of any pandemic.

## Data Availability

The datasets presented in this article are not readily available to protect confidentiality of the participants. Public health workers were exposed to harassment during the COVID-19 pandemic. Requests to access the datasets should be directed to rima-afifi@uiowa.edu.
