# Supplementary material for: Insights from the front line: uplifting stories of the COVID-19 pandemic through the eyes of the public health workforce in Iowa
Source: Front Public Health. 2025 Jul 23;13:1597941. doi: 10.3389/fpubh.2025.1597941 (PMC12325277; doi:10.3389/fpubh.2025.1597941)
Supplement: Supplementary file 1 [file Data_Sheet_1.pdf]

IOWA | PREVENTION RESEARCH CENTERS | IOWA PUBLIC HEALTH ASSOCIATION

Elevating learnings from Iowa's public health system response to COVID-19

You are being asked to participate in this project because individually and within your organizations, you assisted your community to cope with this pandemic in multiple ways. Your experiences are important. This project, a partnership between the University of Iowa's Prevention Research Center for Rural Health and the Iowa Public Health Association, is about uplifting those experiences and stories.

Before you start_. This project uses an innovative approach to gathering data that differs from traditional surveys you may be used to. We will ask you to share an experience. We will then ask short follow-up questions about that experience and about yourself. This should take about 15-20 minutes for you to complete. You can choose not to respond to any question, just select the 'I prefer not to answer' option. Your responses are anonymous so please do not include your names or anyone else's name as you write the story. Also, avoid using words that might identify you or someone else. You are free to skip any questions you prefer not to answer. You are welcome to submit more than one experience/ story and may choose to do so at the end of the survey. Your story will not be shared without your permission even anonymously. It will not be possible to link you to your response on the survey.

If you agree to participate, please click on "I agree" below. If you do not want to participate, please close this page.

Consent

○ ! agree. Thank you again for agreeing to participate!

→

SENSEMAI<ER®

Copyright 2007-2022 Cognitive Edge Pie Ltd. All Rights Reserved. Protected in part by copyright, patent and trademark and other law

Please answer the question below. You can type your answer, or record it if you prefer!

If you want to record audio, simply type "audio" in the open text field below.

11 Imagine you are trying to explain to someone who does not work in public health how our public health system responded to the COVID-19 pandemic in Iowa. What story comes to mind from your own experience that you would share to show what happened? 0

11 You can also record audio rather than write if you'd prefer. If you choose to record, please write N/A in the text box above.

D NIA

12 If you were to give this story a short title, what would it be? 0

13 Now that you have shared your story please let us know how we should take care of your words by choosing one option from below: 0

Q I am appy for my entry to be shored anonymously word-for-word as an example for others to read and to make sense of based on their own experiences

O Please only share my journal entry with the team of researchers/ analysts working with the data

On the triangle shapes below, you will be asked to think about where your story sits between three factors by dragging and dropping a marker onto the shape. You have to click on the marker, or place your finger on it on a touch screen, and drag it to where you want it to be. Even if you want the marker in the centre, you have to move it first in order to activate it.

You can place the marker wherever you like on the triangle. Between corners indicate a mixture of themes or ideas, and the closer the marker is to any of the corners, the stronger that theme or idea is present in this experience.

Below, you will see an example about how that might work with the question of how you spent your day yesterday.

21 In the experience I described...

I hod control over the action

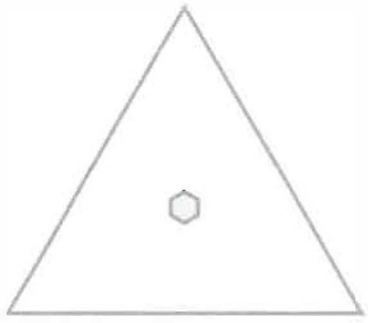

I was able to act

The ability to act was out of my control

O N/A or I prefer not to answer

22 The experience I described was influenced by...

Social, cultural, and economic factors

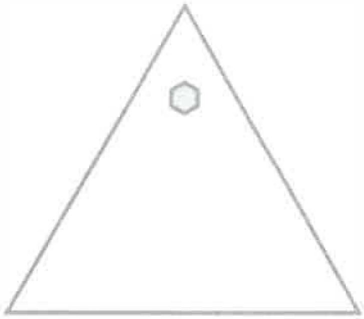

Political decisions

The way things are done around here

O N/A ar I prefer not to answer

2.3 In the experience Idescribed, there was an impact on ...

Myself and my loved ones

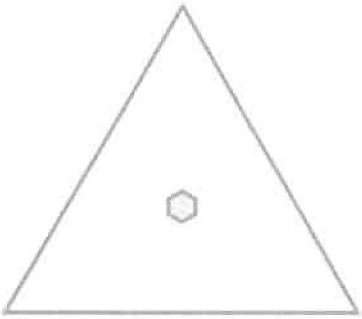

My community

My organization

O N/Aarl prefernottoanswer

2.4 In the experience Idescribed, decisions were made based on...

Organizational values and mission

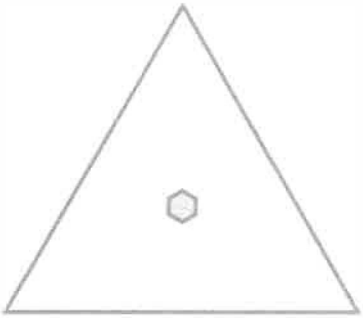

Evidence

Necessity

O N/A or I prefer not to answer

2.5 In the experience Idescribed, responsibility lay with...

Individual actions

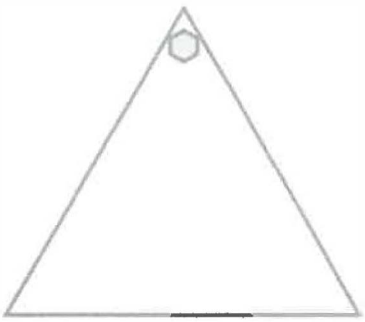

Collective behavior and organization

The government and large organizations

O N/A or I prefer not to answer

2.6 In the experience I described, I noticed people ...

Working with existing partners

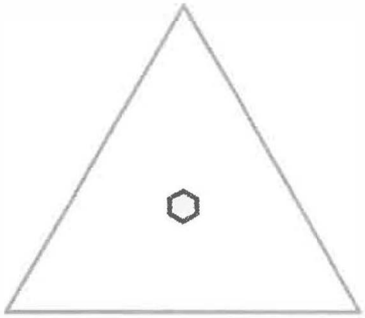

Reaching out to form new local partnerships and relationships

Reaching out to form new notional. state or federal partnerships

O N/A or I prefer not to answer

2.7 In the experience I described, I wished for more...

Access and inclusion

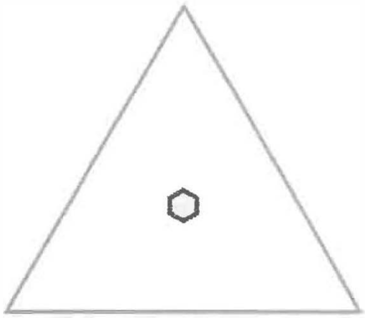

Understanding and empathy

Focus on evidence based public health

O N/A or I prefer not to answer

Now, on the sliding scale below, think about where your story sits between the two endpoints, and move the marker to the appropriate position. If both of the elements are present equally, place your marker in the middle of the sliding scale. The closer the marker is to an endpoint, the stronger the pull of this is for you. You can place the ball anywhere on the slider, if in doubt, go with your gut feel.

3.1 In the experience Idescribed, Ior the public health professionals...

Used tried and tested approaches — Took a risk on something entirely new

O NiA or I prefer not ta answer

3.2 In the experience Idescribed, Ior the public health professionals_

Took the initiative — Waited for instructions

O NiA or I preter notto answer

3.3 In the experience Idescribed, Ior the public health professionals reached out to...

Informal networks — Formal channels

O NiA or I prefer not ta answer

3.4 In the experience Idescribed, public health systems...

Prioritized fast response and action — Prioritized caution and took time to reach a decision

O NiA or I prefer not to answer

3.5 In the experience Idescribed, public health systems...

Reached the some people as before — Reached underserved populations

O NiA or I prefer not to answer

SENSEMAI<ER®

Copyright 2007-2022 Cognitive Edge Pte Ltd. All Rights Reserved. Protecred in part by copyright, potent and t,odemo•k and other law.

Below are a few more questions about the experience you shared earlier. Unless instructed otherwise, please choose one response option only.

4.1. How do you feel about the experience you have shared?

- O Very positive
- Q Positive
- Q Neutral
- O Negative
- Q Very negative
- O I prefer not to answer

O None of these

4.2. Pick up to 3 emotions below that best describe your feelings about your story.

- Amazing
- Angry
- Apathetic
- Appreciated
- Challenged
- Confident
- Excited
- Exhausted
- Frustrated
- Hopeful
- Interested
- Lonely
- Motivated/engaged
- Proud
- Scared
- Safe
- Stressed, worried or anxious
- Unappreciated
- Valued
- Other

- I prefer not to answer

4.3 How quickly did things happen in this experience?

- So quickly there was no time to think
- Quickly
- Average / neither quick nor slow
- Slow and controlled
- Much too slowly
- I don't know
- I prefer not to answer

4.4 During this experience, COVID-19 mitigation strategies (masks, testing, social distancing, etc) were...

0 Available at a cost

0 Not available

0 Available to some

0 Available to all

0 I prefer not to answer

D N/A

SENSEMAI<ER®

Copyrighr 200 -2022 Cognirive Edge Pie Ltd. All Rights Reserved. Protected in part by copyright. patent and trademark and orher lav..

Now, please answer some questions about yourself.

5.1 What feelings best describe your current emotional status related to the pandemic's impact on your work in public health? (pick up to 3)

- D Frustration
- D Uncertainty
- D Hope
- D Feor
- D Anger
- D Helpfulness
- D Confidence
- D Helplessness
- D Other

0 I prefer not to answer

5.1b What other feelings describe your current emotional status?

5.2 What feelings best described your emotional status at the height of the pandemic in Winter 2020-Spring 2021 related to the pandemic's impact on your work in public health? (pick up to 3)

- D Frustration
- D Uncertainty
- D Hope
- D Fear
- D Anger
- D Helpfulness
- D Confidence
- D Helplessness
- D Other

D I prefer not to answer

5.2b. What other feelings described your emotional status at the height of the pandemic?

5.3a During the height of the COVID-19 pandemic in Iowa, how uncertain were you about your own safety?

Very certain	Certain	Neithercertoin nor uncertain	Uncertoin	Very uncertain

O I prefer not to answer

5.3b During the height of the COVID-19 pandemic in Iowa, how uncertain were you about the safety of your family? O

Very certain | Certain | Neither certain nor uncertain | Uncertain | Very uncertoin

O I prefer not to answer

5.3c During the height of the COVID-19 pandemic in Iowa, how uncertain were you about the safety of your community?

Very certain | Certain | Neither certain nor uncertain | Uncertain | Very uncertain

O I prefer not to answer

5.4 What is your outlook and/or how are you feeling abou t the future of public health in Iowa ? (optional)

6.1 How old are you?

- O 18-25
- O 26-35
- O 36-45
- O 46-55
- O 56-65
- O Over65
- O I prefer not to answer

6.2 What is your gender identity? **6.3 What is your gender identify (fill in box_**

- O Woman
- O Mon
- O Non-binary (write in}
- O I prefer not to answer

6.4 Do you identify as transgender?

- O Yes
- O No
- Q I prefer not to answer

6.5 What is your race? (tick all that apply)

D White

O Black or African American

O American Indian or Alaska Native

O Asian

O Native Hawai ion or Other Pacific [slander

O Some other race

O I prefer not to answer

6.6 Are you of Hispanic, Latino or Spanish origin? 0

O Yes

Q No

O I prefer not to answer

6.7 In which Iowa local public health region do you work (select all that apply)? Region 1-6, Statewide

6.8 How would you classify the county(ies) in which you work in terms of population (select all that apply) Rural, Micropolitan, Metropolitan

Nearly there! Please reflect on these short questions. You do not have to answer if you do not want to, and you can answer as many or as few as you'd like.

7.1 Imagine a new but equally virulent pandemic is beginning its spread. Given the experience of the past year and a half, what should we do differently this time in Iowa? (Optional)

7.2 Use the space below to share one insight you've had about the impact of the pandemic in your local community. (Optional)

7.3 How has this process of thinking about your experience felt? (Optional)

Now click on the "SAVE" button below to save your entry! You can also click on the arrow if you need to review your responses.

If you want to share another story, click on the "SUBMIT ANOTHER" button.

←

SAVE

SUBMIT ANOTHER

SENSEMAKER®

Copyright 2007-2022 Cognitive Edge Pte Ltd. All Rights Reserved. Protected in part by copyright, patent and trademark and other law.
