# Supplementary material for: Insights from the front line: uplifting stories of the COVID-19 pandemic through the eyes of the public health workforce in Iowa
Source: Front Public Health. 2025 Jul 23;13:1597941. doi: 10.3389/fpubh.2025.1597941 (PMC12325277; doi:10.3389/fpubh.2025.1597941)
Supplement: Supplementary file 2 [file Data_Sheet_2.pdf]

Supplementary Appendix B

Supplemental Material for "Insights from the front line: Uplifting stories of the COVID-19 pandemic through the eyes of the public health workforce in Iowa"

Interpreting Ternary Diagrams

Compositional data exist when multivariate measurements represent proportions of a whole. As a running example, suppose we measure the proportion of awake time study subjects spend in sedentary behavior, moderate physical activity, and vigorous physical activity. When the whole is divided into three components as in this running example, we may visualize the data through the use of a ternary diagram, or ternary plot. The ternary plot illustrates the data through embedding the three-dimensional compositional variable on an equilateral triangle, where the vertices correspond to the three components. Further, each component also corresponds to a side of the triangle which scales from smaller values away from the vertex to larger values nearer the vertex.

To see how this is done, consider an individual who spends 4/5 (80%) of their time in sedentary behaviors, 2/15 (13.333%) of their time in moderate physical activity, and the remaining 1/15 (6.667%) of their time in vigorous physical activity. Plotting this individual on a ternary diagram yields the following:

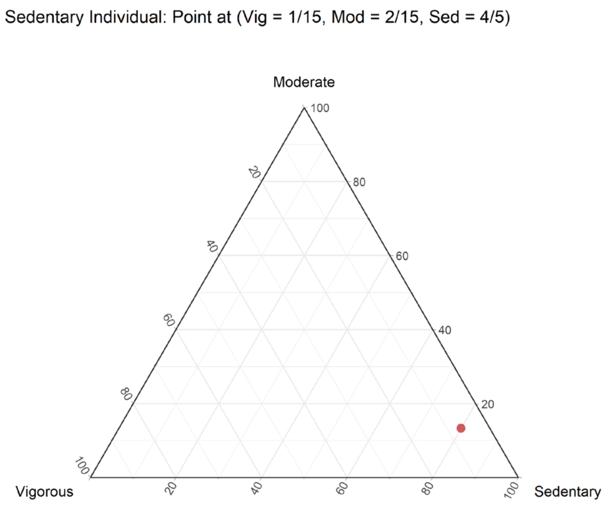

To read the values off of such a plot, we first select a component we wish to focus on, find the edge whose values increase in the direction of the selected vertex, and then draw a line from our point to the component's corresponding side of the triangle. For example, if we

wish to know the percentage of time the individual represented by the red dot in the above ternary plot spends in sedentary activity, see the following plot to obtain 80%:

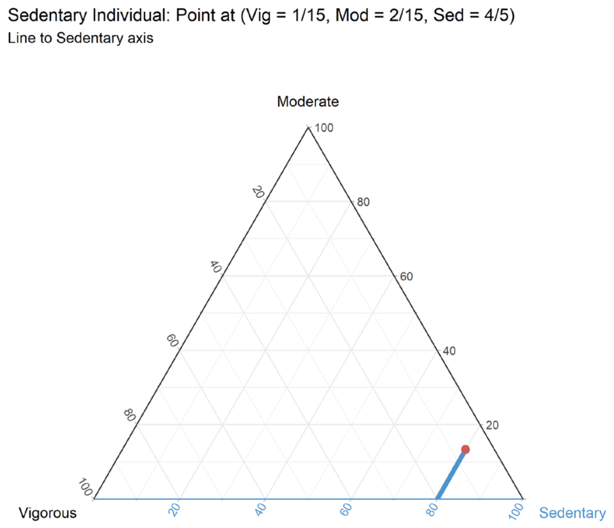

The question then arises as to how that line was drawn. The answer is that the line must be parallel to the edge opposite of the vertex we are focusing on:

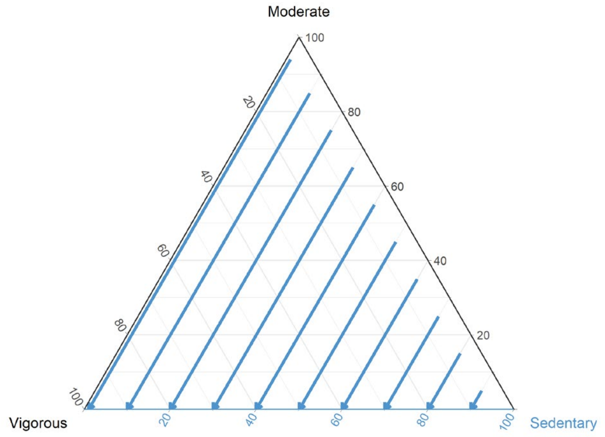

If we want to see what our red point's value of moderate physical activity is, we must again identify the side whose values increase towards the Moderate vertex and draw arrows towards that side which are parallel to the side opposite of the Moderate vertex:

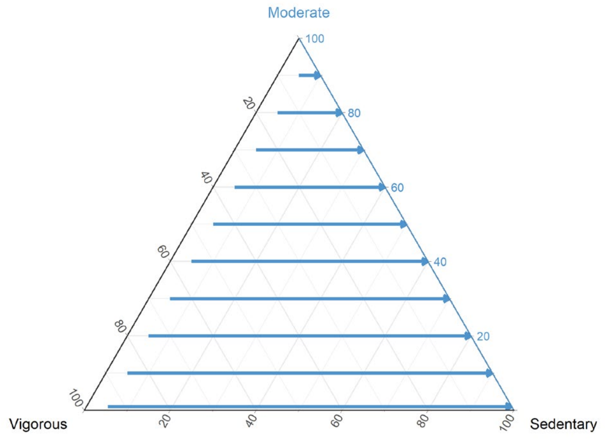

Applied to our example point, we see that our line connects to 13.333% of the individual's time spent in moderate physical activity:

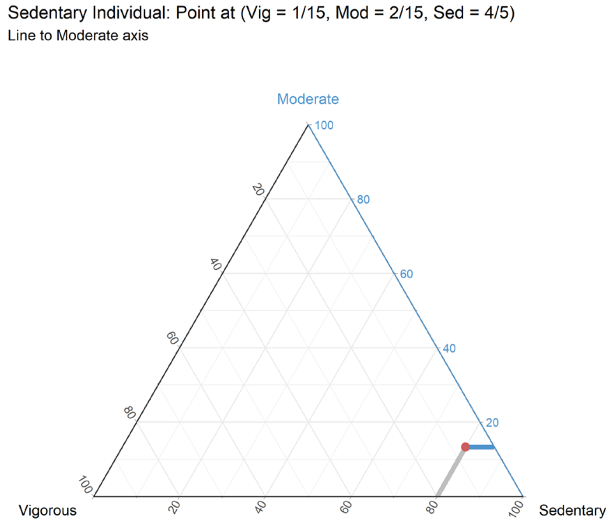

Finally, we can extract the vigorous activity in the same way:

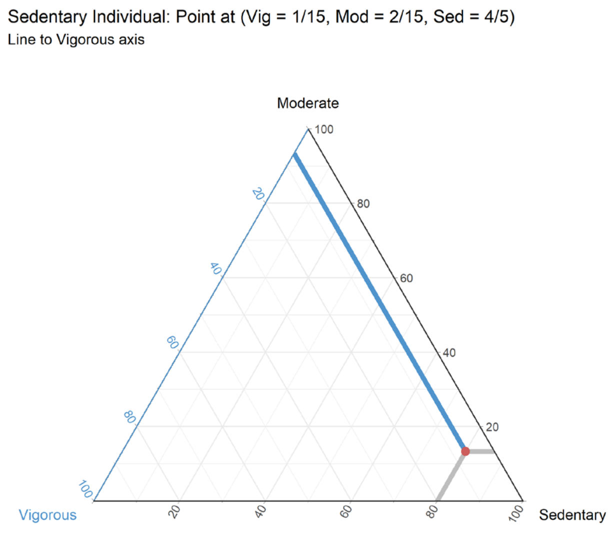

The figures below shows 25 (hypothetical, and not particularly realistic) data points both without and with line segments drawn to highlight the compositional values. A quick look at the scatter of points shows most points nearer the Sedentary vertex, indicating that most of those individuals' time is spent in sedentary activity, and the fact that nearly all points are far from the Vigorous vertex indicates that individuals spend little time in vigorous physical activity.

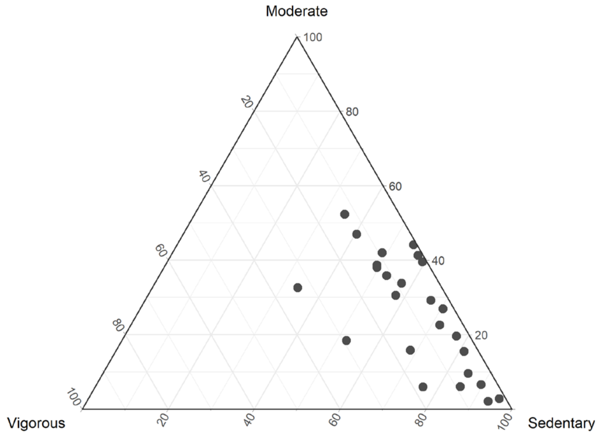

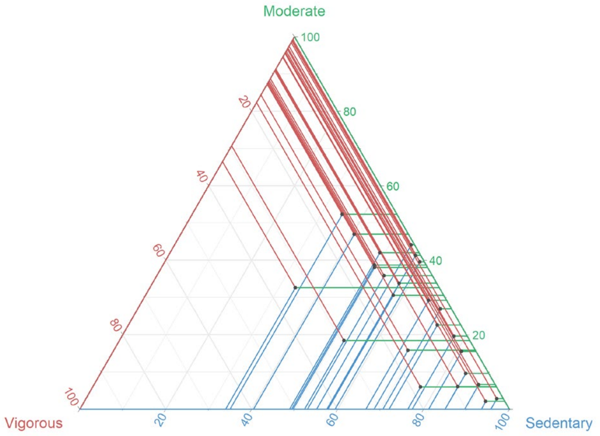

Canonical Correlation Analysis (CCA)

CCA aims to quantify the relationship between two sets of variables through the intuitive measure of Pearson's correlation. Suppose we have n data points and two sets of variables of length q_1 and q_2 respectively. We can store these data in a $n \times q_1$ matrix X_1 and $n \times q_2$ matrix X_2. The goal of CCA is to find two vectors, call them $\boldsymbol{a}$ and $\boldsymbol{b}$ such that $Cor(X_1\boldsymbol{a}, X_2\boldsymbol{b})$ is maximized. That is, we wish to find linear combinations of the $q_1$variables in X_1 and the q_2 variables in X_2 such that there is the highest possible correlation between them. Intuitively, this goal can very loosely be thought of as trying to maximize the correlation between the information in the first q_1 variables and the information in the second q_2 variables. The highest correlation between such linear combinations can be found by obtaining the first singular value of the generalized correlation matrix between X_1 and X_2, i.e., of $\Sigma_{11}^{-\frac{1}{2}}\Sigma_{12}\Sigma_{22}^{-\frac{1}{2}}$, where Σ_{11} is the covariance matrix of X_1, Σ_{22} is the covariance matrix of X_2, and Σ_{12} is the covariance between X_1 and X_2.
