# Supplementary material for: Insights from the front line: uplifting stories of the COVID-19 pandemic through the eyes of the public health workforce in Iowa
Source: Front Public Health. 2025 Jul 23;13:1597941. doi: 10.3389/fpubh.2025.1597941 (PMC12325277; doi:10.3389/fpubh.2025.1597941)
Supplement: Supplementary file 3 [file Data_Sheet_3.pdf]

Supplementary Appendix C: Selected subset of n=10 stories shared by PHW, in response to the prompt "Imagine you are trying to explain to someone who does not work in public health how our public health system responded to the COVID-19 pandemic in Iowa. What would you tell them?" Text is verbatim.

Public Health was given little advance information about a worldwide pandemic that was for the most part not understood and little research had been done due to the new variants. We had to pivot constantly, fight misinformation, and were widely outspoken by the "experts". It was a very tough time for public health with many hours uncompensated and unappreciated. We fought through and did what we individually thought was right for our own counties. We lost friends, colleagues, and some credibility. I still worry what will happen the next time we need people to listen to us!
I worked with Covid Recovery Iowa. In addition to desperate need to help community members learn about epidemiology, biosecurity, and a general understanding disease transmission, our team helped people navigate the challenging feelings associated with the many changes they were forced to adapt to during the pandemic. We helped staff an answer line, and we spent a lot of time helping people put names to the feelings they were experiencing, we shared tools such as deep breathing exercises, noticing thought patterns that were not serving them and helping folks find new ways to connect that felt safe and comfortable to them. We met people where they were, without judgment, to help them holistically through the pandemic. Sometimes we presented at shelters to people experiencing homelessness, other times we joined people in their living rooms, virtually, and teared up with them while they shared about the loved ones or the connections they lost. Each person on our team brought a different expertise, some focusing on mental health, other helping people prepare taxes after a year or more of significant financial changes, others helped business owners keep track of the quickly changing laws. Once the generally community started to feel safe again, we started to help those that had been working on the front line debrief and share about how the previous year had affected them. We shifted our programs to focus on reducing burnout by increasing self-care.
The public health response to COVID-19 in Iowa was multi-faceted. Primarily, it could be broken down into three parts, case investigation/contact tracing, vaccine distribution, and communication/education. While these categories may seem fairly clear, they never before had to be completed at such a large scale, simultaneously, and while guidance was continually shifting. While the public is aware of some of the challenges we faced, many others went unseen. One example would be the state reporting system. COVID-19 lab results from healthcare providers and the state hygienic lab are electronically reported to the state. They then collect those reports and send then electronically alert the appropriate county for follow up. Early on in the pandemic the state reporting system effectively broke. It was not equipped to handle the sheer volume of cases. It became slow, laggy, and would often crash. At one point it was taking someone 2-3 minutes to pull information on a case (when the system was working). Multiply that by 100 cases a day and you are looking at 3-5 hours of someone's time, not to perform investigations or communicate, but to simply access the case information (name, address, phone, etc.). The Iowa Department of Public Health then contracted with a company to build a new system. Public health practitioners had to learn to navigate the new system and incorporate it into their CICT system while still performing all the other necessary work that comes with responding to a pandemic. The new system was an improvement, but like anything new,

came with a different set of problems that had to be fixed or worked around. Innovative solutions had to be created on short notice. The most frustrating part is that this could have been avoided from the start if public health was properly funded and was using adequate and up-to-date technology in the first place. Public health focuses on prevention, and prevention often goes unseen. Awareness of public health activities has never been higher. Hopefully examples like this will serve to increase public health funding on infrastructure so when the time comes to respond to an event such as this we have the tools and systems in place to mount an effective and timely response.
Total confusion. Whether it was news media, State media, or social media, the amount of misinformation and speed at which things changed damaged the reputation of policy makers, medical professionals, and public health agencies all over the US. Furthermore, the damage is still being felt two years later. It seemed the word of every day was "panic" and decisions were made, haphazardly, in response to events as opposed to establishing proactive measures.
Our preparedness for Mass Vaccination clinics through Emergency Preparedness initiatives helped us respond to the vaccination effort. We worked with hospitals, nursing homes, pharmacies, schools, senior health sites, and many other community partners throughout this pandemic. We utilized all agency staff in the response efforts; nurses, home care aide, office staff, management staff, family support workers, environmental staff, dental staff, community health program staff. It was a huge team effort. Besides the pandemic our staff was still continuing to do their normal duties. We utilized other county employees that weren't public health employees i.e., Auditors, , Treasurers office staff, sheriff department, conservation department, etc. Our Board of Health wanted to implement a mask mandate in the county. This did not have a positive response. People were phoning in with their opinions on this, and someone would get very nasty and loud with our staff. During one Board of Health Meeting a number of people phoned in to voice their concerns. Some of the comments were so bad that we decided to keep our agency doors locked out of concern for our staff. There was a segment of the population that believed the pandemic was not real. This population was very negative and hateful on the phone and on social media.
Collaboration, collaboration, collaboration. Our Covid response would not have been as successful as it was without the community collaboration and support. working with all the folks in our community, hospitals, non for profits, medical offices, volunteers and local pharmacies. Contact tracing was tireless, we worked long hard hours, early mornings, late days and nights, worked every weekend.... 7 days a week for months. I myself am on our incident command team and when first heard of Covid-19 was long before others had even known what it was. The changing information and guidance was a lot to keep up with and a few of us were also the go to folks for others with lots of questions. This pandemic has been mentally and physically draining.
The PH response was tricky. We were trying to lead and provide information to the citizens of our county as the information changed rapidly. Often times the community learned information from sources that did not provide accurate data. It felt as though we were fighting a political battle that got in the way of responding to a communicable disease. We were verbally abused on a regular basis when completing phone calls for disease follow-ups and when contacting close contacts for quarantine recommendations. We worked very hard to earn the trust of our county, but rapidly

changing information made it very difficult. The information passed down from IDPH was limited and gave little advance notice for local PH preparation. The community was informed of new information and changes concurrently with local PH. When vaccinations rolled out, we again struggled with limitations for vaccine usage, which further angered community members. We followed the rules very closely for vaccine distribution and were very careful not to waste any doses of vaccine. This took enormous amounts of planning and maneuvering to achieve no vaccine wastage in the early days of distribution. Our phone systems were overwhelmed with phone calls for months. Our local hospital chose not to provide COVID vaccination to the community, which only increased our workload. We did partner with the hospital for some COVID clinics. We provided guidance on COVID to local businesses, churches, city and county government, law enforcement, schools, and healthcare providers. Ultimately, we earned the respect of most of our residents, provided accurate evidence-based data and guidance, and provided COVID vaccinations to all that were seeking them. We also provided vaccinations to those outside our county and state.
COVID-19 was the most challenging piece of my own career. There was so little information, yet it felt like we were getting more information that we could not keep it all straight. The limited availability of vaccine and how it was determined who would get the vaccine was horrific for local workers. Of course you cannot make all happy, but this made it very tough on locals trying to explain the why it was the way it was. We could have used more leadership and explanation from the top. Our local community was scared and some didn't believe in COVID. The politics made everything that much more complicated. Typical responses to things were labeled different and that didn't help those in the position they were in feel good about what they were doing and the why! We all needed to lean on each other and the panic and quick acts of what to do was a now uncharted waters experience. What a ride it has been. Still we feel unknown of what direction to go and when it will be considered an end to it?
Staff from our local public health agency pulled together to make every attempt to prevent or assist with referring community members to obtain testing and medical evaluations when necessary to try to minimize the spread of COVID-19. Every staff member at our agency was delegated some type of duty to help respond to the needs of our community during the pandemic. Clerical staff answered many phone calls routing calls to appropriate staff and mailed out educational material to members of the community. Disease Prevention Specialists, TB Nurses Environmental Health Inspectors and our new team of Contact Tracers made up a strong Contact Tracing Team. Clinic Nurses organized many vaccine clinics both at the health department and around the community. Of course all the coordination was lead by our wonderful management team, epidemiologists, IDPH and the CDC. It was hard work but also very gratifying to answer our communities' questions. As part of the contact tracing team making many phone calls daily to someone newly diagnosed with COVID, I was able to reduce their stress caused by either lack of information or misinformation about COVID.
We went from working on preventative measures to being thrown into the pandemic seemingly overnight when our first cases came in. It was incredible to see how many people across different departments were able to help play a role in our response. People who never work with infectious

diseases came to help those of us who do. We have been living in a constant state of adaptation since then. Everyone has taken on a variety of different roles throughout the last 3 years. One thing everyone has in common is their willingness to help serve and protect the health of Iowans.
