# Supplementary material for: Insights from the front line: uplifting stories of the COVID-19 pandemic through the eyes of the public health workforce in Iowa
Source: Front Public Health. 2025 Jul 23;13:1597941. doi: 10.3389/fpubh.2025.1597941 (PMC12325277; doi:10.3389/fpubh.2025.1597941)
Supplement: Supplementary file 4 [file Data_Sheet_4.pdf]

Supplementary Appendix D rev

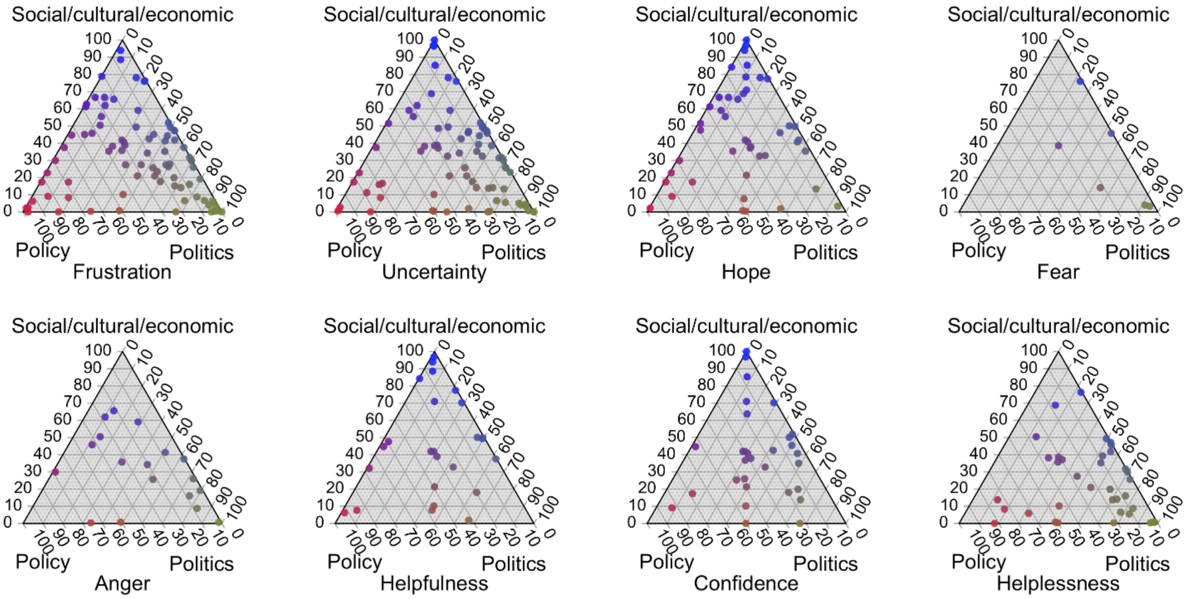

Figure 1: The relationship between public health workers' (PHW) perceived factors influencing their experience and their current pandemic-related emotions; each ternary point represents a unique story. Each plot includes a subset of respondents who expressed the corresponding emotion.

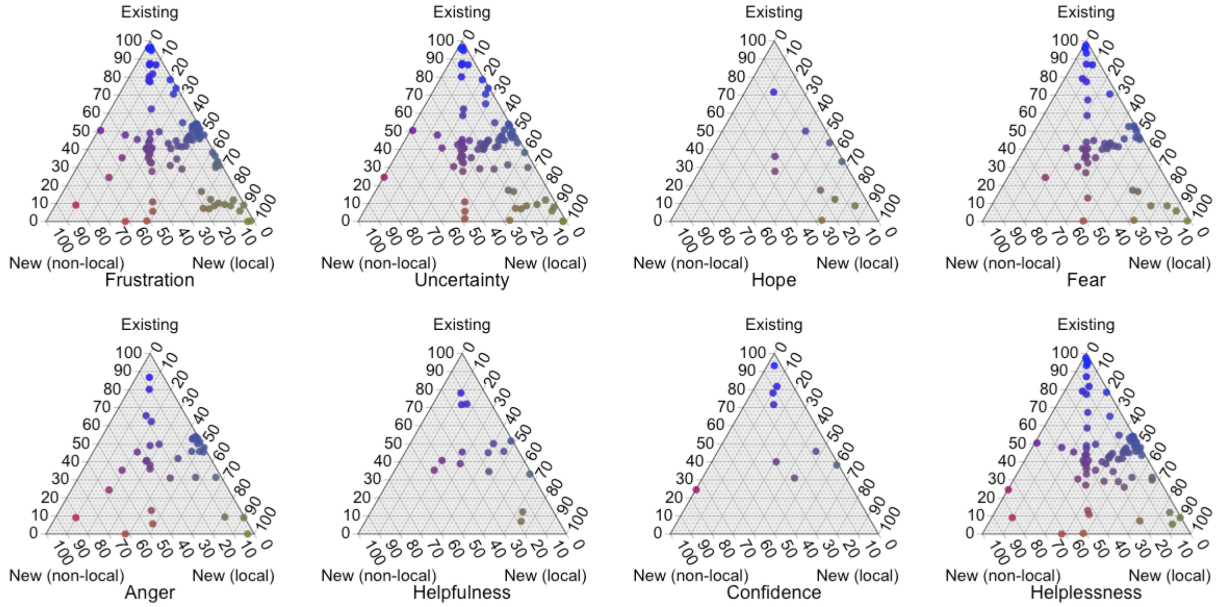

Figure 2: The relationship between public health workers' (PHW) perceived types of collaboration and their feelings at the height of the pandemic; each ternary point represents a unique story. Each plot includes a subset of respondents who expressed the corresponding emotion.

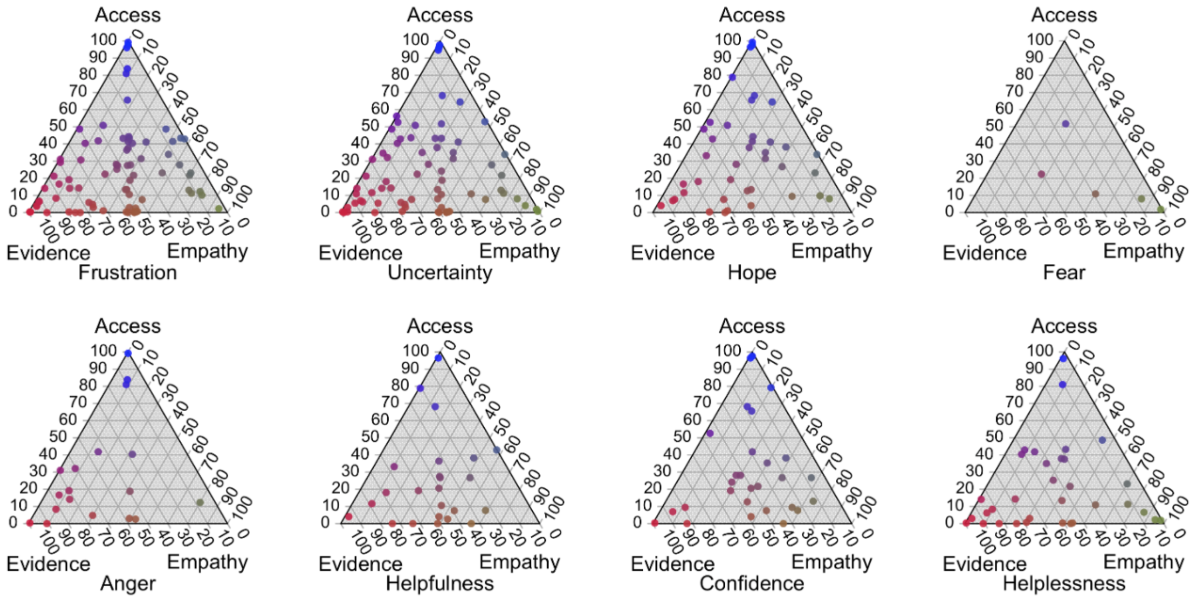

Figure 3: The relationship between public health workers' (PHW) desires or priorities in the context of public health response and their current pandemic-related emotions; each ternary point represents a unique story. Each plot includes a subset of respondents who expressed the corresponding emotion.

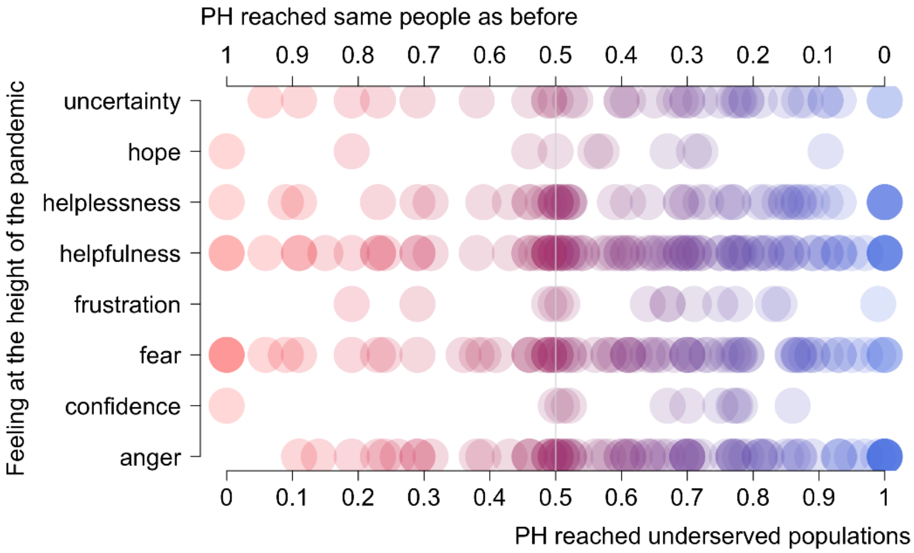

Figure 4: The relationship between public health workers' (PHW) perceived reach and inclusivity of public health efforts and their feelings at the height of the pandemic; each ternary point represents a unique story. Each plot includes a subset of respondents who expressed the corresponding emotion.
